# Dysregulation of decidual NK cell proliferation by impaired decidual cells: a potential contributor to excessive trophoblast invasion in placenta accreta spectrum

**DOI:** 10.3389/fcell.2025.1618461

**Published:** 2025-07-21

**Authors:** You-Zhen Liu, Hsin-Hung Lin, Meng-Shiue Wu, Jin-Chung Shih, Thai-Yen Ling

**Affiliations:** 1 Graduate Institute of Pharmacology, National Taiwan University College of Medicine, Taipei, Taiwan; 2 MediDiamond Inc., Taipei, Taiwan; 3 LuminX Biotech Co. Ltd., Taipei, Taiwan; 4 Department of Obstetrics and Gynecology, National Taiwan University Hospital, Taipei, Taiwan

**Keywords:** decidualization, placenta accreta spectrum (PAS), trophoblast invasion, decidual natural killer cell, immune tolerace

## Abstract

Aberrant interactions among decidual stromal cells, decidual natural killer (dNK) cells, and trophoblasts are implicated in placenta accreta spectrum (PAS) pathogenesis, though the underlying mechanisms remain unclear. This study investigates the relationship between defective decidualization of endometrial stromal cells and dysregulated dNK cell proliferation, which may contribute to excessive trophoblast invasion and the development of PAS. We established an *in vitro* system that mimics the decidual microenvironment to investigate these interactions. Maternal decidua-derived mesenchymal stem cells (MD-MSCs) from healthy pregnancies and PAS patients (PA-MSCs) were isolated and induced to undergo decidualization using hormonal and chemical stimuli. Peripheral natural killer (pNK) cells were then co-cultured with these MSCs to generate dNK-like cells. Cellular interactions among MSCs, dNK-like cells, and trophoblasts were evaluated using an *in vitro* co-culture system. Decidualization defects in PA-MSCs were marked by reduced morphological changes and dysregulated expression of decidual markers, potentially associated with estrogen receptor (ER) overexpression. Furthermore, both PA-MSCs and normal MD-MSCs similarly regulated trophoblast invasion, suggesting an indirect impact of impaired decidual cells on trophoblast behavior. Interestingly, decidualized MD-MSCs (De-MD-MSCs) showed the potential to induce the conversion of pNK cells into dNK-like cells, which displayed reduced cytotoxicity on trophoblasts and elevated KIR2DL4 expression. These dNK-like cells exhibited increased proliferation when co-cultured with PA-MSCs, enhancing trophoblast invasion and spiral artery remodeling. Conditioned medium derived from PA-MSCs-induced dNK-like cells demonstrated a higher capacity to promote trophoblast invasion in a dose-dependent manner. The abnormal proliferation of dNK cells induced by impaired decidual cells may contribute to the pathogenesis of PAS, providing valuable insights into its mechanisms and potential therapeutic interventions.

## Introduction

In the process of embryos implanting in the uterus, the interaction between the maternal decidual cells, leukocyte subpopulations like decidual natural killer (dNK) cells, and blastocyst trophoblasts is critical for healthy placental formation and successful pregnancy (Xu et al., 2017). During blastocyst development, trophoblasts differentiate into cytotrophoblasts (CTBs), the subsequent syncytiotrophoblasts (SCTs), and extravillous trophoblasts (EVTs), forming the primitive syncytium and lacunae (Kim et al., 2024; Kojima et al., 2022). Studies demonstrate that decidual and dNK cells regulate trophoblast invasion by secreting factors, maintaining a delicate balance (Yang et al., 2019). During pregnancy, decidual cells, part of the specialized endometrium, complete the decidual reaction post-implantation, supporting placentation through interactions with trophoblasts and producing specific proteins and factors (Hess et al., 2007). Throughout pregnancy, dNK cells, with unique killer cell immunoglobulin-like receptors (KIR) expression and interactions with decidual cells, play a crucial role in immune tolerance at the maternal-fetal interface and placentation through interactions with EVTs (Jabrane-Ferrat, 2019). The equilibrium among decidual, dNK, and trophoblast cells is vital in establishing an immune-regulated environment necessary for placental formation and fetal development, contributing significantly to maternal-fetal immune tolerance (Wang and Li, 2020; Hanna et al., 2006).

Various placental disorders, including the high-risk placenta accreta spectrum (PAS), are caused by disruptions in the regulation between these cells (Al-Khan et al., 2013). PAS is an abnormal placental development condition that encompasses placenta accreta, increta, and percreta and is a major cause of severe maternal morbidity and mortality (Bartels et al., 2018). PAS is particularly daunting for pregnant women and obstetricians due to the difficulty in early detection and the complexity of treatment even after diagnosis. The prevalence rate of PAS has increased in recent decades, from 1 in 1,250 births in the 1980s to as many as 1 in 272 births today (preventaccreta.org, 2024). From the cytological perspective, studies suggest that defects in the decidualization of endometrial stromal cells (ESCs) (Illsley et al., 2020; Gao et al., 2022), altered immune responses in maternal tissues (Adu-Gyamfi et al., 2021; Mirani et al., 2024), or a combination of both conditions (Velicky et al., 2016; Sharma et al., 2016; Moffett and Shreeve, 2023) are proposed as underlying factors contributing to excessive invasion of EVT. This can result in the pathologic adherence of the placenta and subsequent severe bleeding during delivery (Wang et al., 2021; Chang et al., 2020; Murata et al., 2022). However, due to the lack of appropriate animal models and even cellular models, studying the intricate mechanisms governing the aberrant placentation of PAS has presented challenges. Thus, the precise etiology of PAS remains incompletely understood, especially when investigating the early and mid-stages of human pregnancy, during which EVTs interact with decidual cells and dNK cells. Therefore, understanding the complex interactions among decidual cells, EVTs, and immune cells, particularly dNK cells within the uterus, is essential for developing diagnostic and therapeutic approaches to address PAS and its potentially life-threatening complications.

In our preliminary work, we isolated a unique cell population derived from the choriodecidual interface, where the decidua (developed from the endometrium) and the chorion (created from the trophoblast of the outer wall of the embryo) fuse. In order to clearly demonstrate the basic characteristics of MSCs, we adapted the results from previous studies (Su et al., 2017). These cells were identified from the precise decidua with the MSC markers (Supplementary Figure S1A) and differentiation potentials (Supplementary Figure S1B), known as human placenta maternal decidua-derived mesenchymal stem cells (MD-MSCs), replacing the previously confusing origin and nomenclature of human placenta choriodecidual membrane-derived mesenchymal stem cells (pcMSCs) (Su et al., 2017). They have shown significant potential for cell therapy due to their anti-inflammatory, immunomodulatory, and tissue repair capabilities (Chen et al., 2022). Beyond their roles in regenerative medicine, this study aims to explore the physiological functions of MD-MSCs in the endometrium to gain new insights into complex placental diseases. Consequently, we developed an MD-MSC-based decidualization and co-culture system. This advanced system facilitates the assessment of EVT invasion and dNK cell induction, thereby overcoming the limitations of traditional *in vitro* methods.

In this study, we established an MD-MSC-based co-culture system and utilized pathological MD-MSCs from patients with PAS (PA-MSCs) to investigate the underlying mechanism of PAS. Our findings suggest that MD-MSCs can potentially induce pNK cells to convert into dNK-like cells with lower cytolytic ability and enhanced expression of KIR2DL4, one of the polymorphic KIRs that interacts with HLA-G on EVTs (Rajagopalan and Long, 2012). Moreover, we observed that dNK-like cells proliferate significantly when co-cultured with PA-MSCs and exhibit a greater capacity to promote trophoblast invasion when co-cultured with MD-MSCs. This discovery highlights the potential contribution of aberrant dNK cell numbers and decidualization defects to PAS development. This study proposes a novel approach to studying the intricate interactions among decidual, dNK, and trophoblast cells *in vitro* and offers new insights into the mechanism of PAS.

## Materials and methods

### Isolation and culture of MD-MSCs and PA-MSCs

MD-MSCs and PA-MSCs cells were obtained from the term placenta undergoing cesarean sections at Taipei Medical University Hospital and Dr. Jin-Chung Shih from National Taiwan University Hospital, respectively. All procedures were approved by the Institutional Review Board (IRB: 202308101RINC). The diagnostic criteria for selecting pathological placentas as clinical specimens in this study are outlined in Supplementary Table S1. Additionally, histopathological confirmation of PAS was performed, with Supplementary Figure S4 illustrating interstitial cytotrophoblast (iCTB) invasion into the myometrium in the three selected specimens. For cell isolation, the decidual membrane was isolated, chopped, and digested with a mixture of protease (Sigma, 9001-92-7), collagenase B (Sigma, COLLB-RO), and DNase I (Bioshop, DRB003). After overnight digestion at 4°C, the filtrate was centrifuged, washed, and resuspended in MCDB201 medium (Merck, M230428) supplemented with 1% insulin-transferrin-selenium (ScienCell, 0803), 10 ng/mL epidermal growth factor (EGF, Peprotech, AF10015), and 1% penicillin/streptomycin. Cells were seeded onto collagen-type IV-coated Petri dishes, and non-adherent cells were removed after 24 h. Upon reaching 90% confluency, cells were dissociated with trypsin (Gibco, 25-200-072), terminated with fetal bovine serum (FBS) (Gibco, 10-082-147), and seeded for further amplification.

### *In vitro* decidualization

For *in vitro* decidualization using (a) hormone and (b) second messenger induction, MD-MSCs or PA-MSCs were initially seeded in a 10-cm Petri dish and allowed to attach for 48 h. Subsequently, the cells were stimulated with the combination of (a) 10 nM estradiol (E2, Sigma-Aldrich, E2785) and 1 μM progesterone (P4, Sigma-Aldrich, P8783) or (b) 0.5 mM 8-Bromo-cAMP sodium salt (MedChemExpress, HY-12306) and 1 μM medroxyprogesterone 17-acetate (MPA, Sigma-Aldrich, M1629) for 5 days. The morphology and the expression of decidualization markers were assessed at 5 days following induction. Throughout the decidualization process, the morphological changes of the cells were monitored using a phase-contrast microscope on the fifth day after induction. Furthermore, for further analysis, total protein and RNA were extracted from decidualized cultures and non-stimulated control samples at specified time points.

### Decidual cell morphology quantification

MD-MSCs were initially seeded in a sterilized 12-well plate and subjected to two different decidualization cocktails. Following a 5-day decidualization period, cells were washed with PBS (pH 7.4) and fixed with 4% paraformaldehyde solution (BioLegend, 420801) in PBS for 10 min at room temperature. After fixation, cells underwent three PBS washes. They were stained with Wheat Germ Agglutinin (WGA) (Invitrogen, W11261) at a concentration of 5 μg/mL to target the cell membrane, enhance the visibility of cell boundaries, and facilitate the cell morphology quantification. For nuclear visualization, DAPI (BioLegend, 422801) was applied at a concentration of 1 μg/mL for 5 min. Stained cells were then observed using fluorescence microscopy to capture images, and ImageJ software was employed to quantify cell morphology. The circularities and roundness of 15 cells from each image were measured separately to assess cell shape, ensuring reliable statistical analysis.

### Real-time PCR (RT-PCR)

Total RNA was extracted from MD-MSCs and PA-MSCs using the NucleoSpin® RNA isolation kit (740955.50, Macherey-Nagel). The concentration of RNA was determined using the Nanodrop 1000 (Thermo) spectrophotometer. Subsequently, reverse transcription was performed using the SuperScript first-strand synthesis system (Invitrogen, 48190-011) according to the manufacturer’s instructions. The resulting cDNA samples were utilized for PCR amplification using the qPCRBIO SyGreen Blue Mix Lo-ROX (PB20.15). Quantitative PCR (qPCR) assays were conducted in triplicate on a Biometra Professional Basic 96 gradient detection system. To normalize the expression levels of target genes, the reference gene (18S rRNA) was used. The specific primers employed for each gene can be found in Supplementary Table S2. The average threshold cycle (Ct) was calculated from the Ct values obtained from three replicates of each sample. The normalized ΔCt value for each sample was computed as the mean Ct of the target gene minus the mean Ct of the reference gene. ΔΔCt was then calculated as the difference between the ΔCt values of the control and test samples. The fold change in gene expression for each sample relative to the control was determined using the 2^(−ΔΔCt)^ mathematical model for relative quantification in qPCR. The mean fold induction and standard error of the mean (SEM) were determined from a minimum of three or more independent experiments.

### Enzyme-linked immunosorbent assay (ELISA)

To measure the concentration of secreted proteins, such as prolactin (PRL) and Insulin-like growth factor binding protein 1 (IGFBP1), the cell culture medium was collected and centrifuged at 2,000 g for 10 min at 4°C to remove any cellular debris. The resulting supernatant was immediately supplemented with a protease inhibitor cocktail and stored at −80°C for subsequent analysis. For the determination of scavenger receptor class A type 5 (SCARA5) protein levels, the cells were first lysed using RIPA buffer supplemented with a 100-fold diluted protease inhibitor cocktail. The cell lysate was then collected and centrifuged at 16,500 g for 20 min. The resulting supernatant was transferred to a new Eppendorf tube and stored at −80°C until further use. To quantify the PRL and IGFBP1 secretion levels, specific ELISA kits were employed, such as the Human Prolactin DuoSet ELISA (R&D #DY682) and Human IGFBP1 DuoSet ELISA (R&D #DY871), in conjunction with the DuoSet ELISA Ancillary Reagent kit 2 (R&D #DY008B), following the manufacturer’s instructions. Similarly, the SCARA5 levels were determined using an appropriate ELISA kit (AVIVA SYSTEMS BIOLOGY, OKCD00526).

### Immunohistochemistry (IHC) in tissue sections

The human decidual tissue sections from normal and PAS placenta were formalin-fixed, paraffin-embedded, cut, and mounted at the Laboratory Animal Center, College of Medicine, National Taiwan University. The dissected sections were placed in a 60°C oven to soften the paraffin for the following steps. After 30 min, the sections were dewaxed with Xylene-clear citrus oil (National Diagnostics, HS-200), cleared in 100% ethanol, and rehydrated through gradients of ethanol in PBS (with gradients of 100%, 95%, 80%). Antigen retrieval was then conducted in 100°C citrate acid buffer for 20 min. Sections were sequentially incubated with 1% blocking BSA for 5 min and subsequently incubated with primary antibodies at 4°C overnight. After washing with PBS to remove unbound antibodies, biotinylated secondary antibodies were applied. Following that, HRP polymer was applied for 30 min at room temperature. After 30 min, diaminobenzidine (DAB) substrate (Chromogen: Substrate = 1:20) was added for precipitation for 1–3 min. Hematoxylin was then used to stain cell nuclei. Sections were mounted with Malinol (MUTO PURE CHEMICALS, 2040-2). The information on the antibodies used is provided in Supplementary Table S3. IHC staining reagents were used following the manufacturer’s instructions (Novolink, RE7290-k).

### Surface marker expression levels measured by flow cytometry

For the evaluation of ER, PR, and SCARA5 expression levels in MD-MSCs and PA-MSCs, live cells were trypsinized and fixed with a 4% paraformaldehyde solution (BioLegend, 420801) in PBS for 10 min at room temperature. Permeabilization was achieved using 0.1% Triton X-100 for 5 min at room temperature. Subsequently, fixed cells were incubated individually with ER, PR, and SCARA5 antibodies at manufacturer-specified concentrations for 15 min. For surface CD3, CD56, KIR2DL4, and HLA-G expression levels, the same protocols were followed without Triton X-100 permeabilization. Finally, cells were resuspended in FACS buffer (1X PBS supplemented with 1 mM EDTA, 25 mM HEPES, and 1% FBS) for subsequent analysis. Analytical flow cytometry was performed using an LSRFortessa, and FACS data were analyzed using FlowJo (version 10.8.01).

### Trophoblast invasion assay

To perform the invasion assay, MD-MSCs and PA-MSCs were seeded and cultured in a 24-well plate. Subsequently, these cells underwent treatment with two distinct decidualization cocktails. Following a 5-day decidualization, trophoblast cell line 3A-sub-E (BCRC, 60302) was seeded onto transwell inserts with an 8 μm pore size to facilitate trophoblast invasion. Many mammalian cells can deform and squeeze through 8 μm pores, making this size appropriate for invasion studies. These inserts had been pre-coated with a Matrigel solution (Corning, #356231) diluted sixfold in serum-free MEM α (Minimum Essential Medium) (Gibco, 11900024). After a 3-day co-culture period, the transwell inserts were meticulously removed, and non-invaded cells present on the upper surface of the inserts were gently eliminated using a cotton swab. Subsequently, the invaded trophoblasts were lysed utilizing a CellTiter-Glo® 2.0 Cell Viability Assay (Promega #G9241) to facilitate the quantification of the relative trophoblast invasion rate.

### Peripheral blood mononuclear cells (PBMCs) isolation and peripheral blood natural killer (pNK) cells purification

pNK cells were obtained from human peripheral blood following approved procedures by the IRB (202312090RINC). Mononuclear cells were isolated from human peripheral blood using the Mitenyl Biotec protocol for density gradient centrifugation. Peripheral blood was collected in a 10 mL vacutainer containing heparin and centrifuged at 550 g for 10 min to separate plasma from blood cells. The plasma was inactivated at 56°C for 30 min, followed by centrifugation at 3,000 g for 10 min to remove precipitate. For PBMC isolation, blood cells were diluted with four times the volume of commercial 1X PBS (Gibco, 00187) and layered over Ficoll-Paque (Cytiva, 17144002) in a 50 mL conical tube, then centrifuged at 400 g for 40 min. The PBMC layer was carefully transferred to a new tube, washed with 1X PBS, and centrifuged at 300 g for 10 min. Platelets were removed by resuspending the cell pellet in 1X PBS and centrifuging at 200 g for 15 min, repeating the step twice. The resulting cell pellet was resuspended, and a cell count was performed. A total of 10^7^ cells were seeded in a T75 flask and cultured in a DSNK medium (BioMab, 020-A010-001) supplemented with inactivated plasma for pNK cell purification and expansion. After a 14-day selection period, the purity of pNK cells was assessed by flow cytometry by determining the expression of the pNK cell marker CD3^−^CD56^+^. Once purity reached 95%, pNK cells were considered suitable for subsequent experiments.

### Cytotoxicity assay for pNK cell and dNK-like cells

The pNK cells and dNK-like cells induced by MD-MSCs were prepared according to the aforementioned protocols. In the cytotoxic assay, 3A-subE trophoblasts were initially seeded into a 96-well plate. Following a 6-h attachment period, the pNK cells and dNK-like cells were seeded to co-culture with the attached trophoblasts. The ratios of effector cells (NK cells) to target cells (trophoblasts) were set at five different proportions: 1:1, 2:1, 4:1, 1:2, and 1:4. After co-culturing for 12 h, the NK cells were removed with the cell culture medium, and the remaining attached trophoblasts were lysed using the CellTiter-Glo® 2.0 Cell Viability Assay and measured for luminescence. The cytolytic ability of NK cells was quantified using the formula:

$$\left(\frac{Luminescenceendpointof\left(Trophoblastonlygroup–NKwithtrophoblastgroup\right)}{Luminescenceendpointoftrophoblastonlygroup}\right)\times 100\%$$.

### dNK-like cell proliferation assay

MD-MSCs and PA-MSCs were seeded in 96-well plates at a density of 5 × 10^3^ cells/well and cultured for 48 h. These cells were then treated with two different decidualization cocktails. Following a 5-day decidualization period, purified pNK cells were seeded at a density of 5 × 10^3^ cells/well into the 96-well plates, establishing co-cultures with MD-MSCs, PA-MSCs, and the decidual cells. During the co-culture period, the suspended NK cells were collected at specific time points: 1, 4, 7, and 10 days. To quantify the proliferation of dNK-like cells, the CellTiter-Glo® 2.0 Cell Viability Assay (Promega #G9241) was employed. This assay allows measurement of cell viability based on ATP levels, providing insights into cell proliferation and overall cellular health.

### RNA-sequencing and data analysis

MD-MSCs and PA-MSCs were cultured in 10 cm Petri dishes. Following treatment with various decidualization cocktails, the total RNA of these cells was extracted using the NucleoSpin® RNA isolation kit (740955.50, Macherey-Nagel) and subsequently utilized for RNA-seq analysis. The RNA-seq procedure was carried out by BIOTOOLS (Taiwan). The genetic data obtained was analyzed using the Biotools RNAseq version 1.6.6 platform. This analysis yielded fold changes, Log_2_ (fold changes), and p-values for each gene, allowing for the identification of differentially expressed genes (DEGs) based on Log_2_ (fold changes) > 1 and p-values <0.05. The values presented on the heatmap are expressed as z-scores, which have been normalized relative to the gene expression levels. Gene expression plots were generated using the website SRplot, while Venn diagrams were created using Venny2.0.

### Statistical analysis

Statistical analysis was performed using GraphPad Prism version 9.1.1 (GraphPad Software, La Jolla, CA, United States). The data were presented as mean ± SEM (standard error of the mean). Statistical comparisons were conducted using one-way ANOVA with Dunnett’s *post hoc* correction or two-way ANOVA followed by Tukey’s or Bonferroni’s *post hoc* tests. A significance level of P < 0.05 was considered statistically significant (*P < 0.05, **P < 0.01, ***P < 0.001, n.s.: no significance).

## Results

### MD-MSCs represent a distinct cell type with the capability for *in vitro* decidualization

**FIGURE 1:**
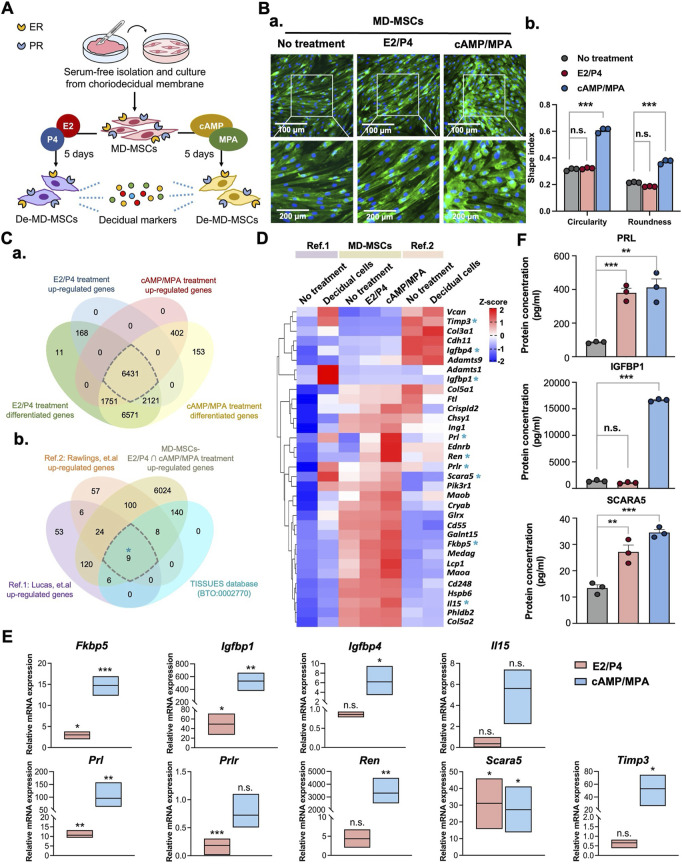
Characterization of MD-MSCs for decidualization potential through morphological changes and identification of decidual cell markers. **(A)** Schematic representation of the workflow for MD-MSCs isolation and culture for subsequent analysis. **(B) (a)** Representative images of normal MD-MSCs stained with WGA under E2/P4 and cAMP/MPA treatments. **(b)** Quantification of changes in circularity and roundness in normal MD-MSCs following decidualization treatment, assessed using ImageJ software. **(C) (a)** Venn diagrams display differentially upregulated genes’ overlap in both E2/P4 and cAMP/MPA treatment groups. **(b)** A selection of 6431 upregulated genes was subsequently overlaid with two published databases to identify 33 potential decidual cell markers. 9 annotated genes are selected from the upregulated genes in decidual cells within the curated database TISSUES (BTO:0002770). **(D)** Heatmap presenting the expression levels of the 33 annotated upregulated genes during decidualization in MD-MSCs and two reference databases. Values are expressed as z-scores, which have been normalized to the gene expression levels. The asterisk key “*” signifies the annotation of 9 genes for potential validation as decidual cell markers. **(E)** Validation of 9 selected genes through qPCR analysis. *Prl*, *Igfbp1,* and *Scara5* are identified as potential marker genes for De-MD-MSCs. Values are normalized to **(F)** The protein expression levels of PRL, IGFBP1, and SCARA5 of MD-MSCs under E2/P4 and cAMP/MPA decidualization treatment. Values are expressed as mean ± SEM. *p < 0.05, **p < 0.01, and ***p < 0.001. *Prl*: Prolactin; *Igfbp1*: Insulin-like growth factor binding protein 1; *Scara5*: Scavenger receptor class A type 5.

In this study, we have discovered a particular variety of MSCs known as MD-MSCs. These cells were meticulously extracted from the decidual membrane of the human placenta, a process accomplished through serum-free culture techniques. In addition, they displayed a remarkable capacity for amplification *in vitro*, with the ability to undergo approximately 20 passages (Su et al., 2017). Given their inherent expression of estrogen (ER) and progesterone receptors (PR), we hypothesized that they could respond to hormonal stimuli and undergo decidualization (Figure 1A).

To thoroughly assess the efficacy of the transition from MD-MSCs to decidual cells, we employed two well-established metrics: alterations in cell morphology and the upregulation of specific genes. Following a 5-day induction period with hormonal (E2/P4) or chemical (cAMP/MPA) agents (Figure 1A), MD-MSCs underwent significant morphological transformations, transitioning from a spindle-like to a rounded shape, as illustrated in Figure 1B-a. To quantify these observed changes in shape, we applied circularity and roundness parameters (Figure 1B-b) (Pan-Castillo et al., 2018). Remarkably, upon exposure to the second messenger induction, three distinct batches of MD-MSCs exhibited a pronounced shift in morphology (Supplementary Figure S2A).

To further investigate the genetic changes associated with decidualization in MD-MSCs, we conducted an RNA-seq analysis to explore potential decidual cell markers. We compared our dataset with two previously published single-cell RNA-seq databases (Lucas et al., 2020; Rawlings et al., 2021), aiming to identify upregulated genes that could serve as reliable biomarkers for decidual cells. This analysis resulted in the identification of 33 annotated genes (Figure 1C), the expression patterns of which were then visualized in heatmap format, as depicted in Figure 1D; Supplementary Data 1. Furthermore, 9 annotated genes are further investigated as potential decidual cell markers through comparison with the TISSUES database (BTO:0002770) (Supplementary Data 1). To validate the results obtained from RNA-seq, we conducted qPCR analysis on these 9 annotated genes (Figure 1E). Notably, three genes including prolactin (*Prl*), insulin-like growth factor binding protein 1 (*Igfbp1*), and scavenger receptor class A type 5 (*Scara5*) emerged as potential biomarkers for decidual cells. While IGFBP1 showed clear upregulation at both the transcript level, as confirmed by qPCR, and the protein level by ELISA, its differential expression appeared less prominent in the heatmap (Figure 1D). This discrepancy is likely due to the normalization method used in the transcriptomic analysis. Global scaling across all conditions can mask relative fold changes, especially when gene expression is compared against highly expressed reference transcripts (Supplementary Figure S3). As a result, genes that are genuinely upregulated may appear less visually distinct in the heatmap representation. Furthermore, the potential decidual cell markers were selected based on a 10-fold change observed in both E2/P4 and cAMP/MPA treatments to avoid the induction of nonspecific upregulation through chemical stimulation. Previous studies have shown that MSCs from either bone marrow or adipose tissue could also undergo decidualization through cAMP/MPA treatment. However, it’s important to note that the cAMP signaling pathway can bypass the ER and PR, directly driving downstream processes and promoting nonspecific signal transduction (Yoshie et al., 2015). To address this concern, we also employed other types of MSCs to distinguish the decidualization potential of MD-MSCs. As anticipated, following E2/P4 treatment, the mRNA expression levels of *Prl*, *Igfbp1*, and *Scara5* were dramatically upregulated in MD-MSCs, compared with bone marrow-derived MSCs (BMMSCs) and adipose tissue-derived MSCs (Supplementary Figure S2B). Furthermore, the protein expression levels of these three decidual cell markers measured by ELISA were consistent (Figure 1F).

### Decidualization defects in PA-MSCs have been observed in the MD-MSC-based decidualization system

**FIGURE 2:**
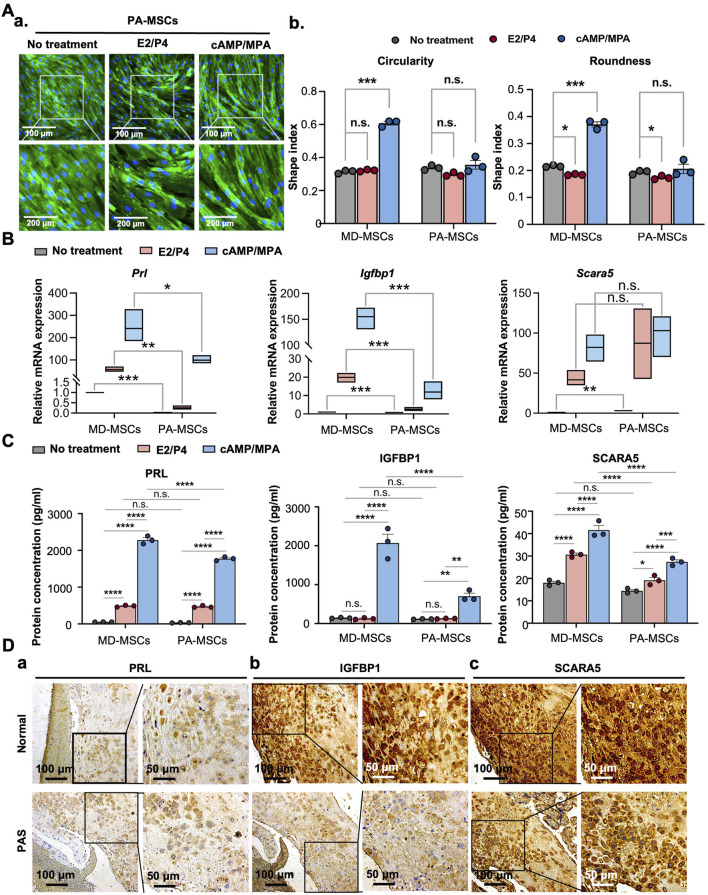
PA-MSCs demonstrate decidualization defects with reduced morphological changes and decidual cell marker expression levels. **(A) (a)** Representative images and quantification of **(b)** circularity and roundness changes in PA-MSCs stained with WGA under E2/P4 and cAMP/MPA treatments compared with MD-MSCs. **(B)** The mRNA expression levels of three potential decidual cell markers, *Prl*, *Igfbp1*, and *Scara5*, in PA-MSCs compared with MD-MSCs. **(C)** The protein expression levels of three potential decidual cell markers, PRL, IGFBP1, and SCARA5, in PA-MSCs compared with MD-MSCs. **(D)** Immunohistochemistry images showing the differential expression levels of **(a)** PRL, **(b)** IGFBP1, and **(c)** SCARA5 in decidual cells between normal and PAS patients. Values are expressed as mean ± SEM. *p < 0.05, **p < 0.01, and ***p < 0.001. PRL: Prolactin; IGFBP1: Insulin-like growth factor binding protein 1; SCARA5: Scavenger receptor class A type 5.

Building upon previous research providing solid evidence for the decidualization potential of MD-MSCs, we aimed to delve deeper into the mechanisms underlying PAS, a condition associated with decidualization defects (Hecht et al., 2020). By utilizing PA-MSCs, we sought to unravel the detailed mechanisms of this disease through the decidualization system. We hypothesized that PA-MSCs might exhibit an inadequate degree of decidualization, rendering them unable to effectively resist trophoblast over-invasion (Aplin et al., 2020). First, as the representative images showed, we observed that PA-MSCs displayed fewer morphological changes compared to MD-MSCs (Figure 2A-a). Quantitative analysis revealed that PA-MSCs retained their spindle shape rather than adopting a round shape, as indicated by their low roundness and circularity values (Figure 2A-b). Additionally, we assessed the mRNA (Figure 2B) and protein (Figures 2C,D) expression levels of three annotated decidual cell marker genes in PA-MSCs. As shown in the figures, the mRNA expression levels of *Prl* and *Igfbp1* were significantly reduced in decidualized PA-MSCs (De-PA-MSCs) compared to De-MD-MSCs. Although there were no significant differences in protein levels between De-PA-MSCs and De-MD-MSCs by ELISA, immunohistochemistry staining of placental tissues revealed significantly reduced expression of PRL and IGFBP1 proteins in PAS samples compared to normal placenta. These findings indicate that the downregulation of *Prl* and *Igfbp1* at the mRNA level is reflected at the protein level in placental tissues, supporting a consistent trend across molecular and histological analyses. Conversely, the gene expression levels of *Scara5* were higher in De-PA-MSCs but reduced in protein levels. The inconsistency between SCARA5 mRNA and protein expression levels is likely attributable to post-transcriptional and translational regulation, which can modulate protein abundance independently of transcript levels. Nevertheless, these observations indicate a clear distinction in gene expression between PA-MSCs and MD-MSCs, suggesting a defective decidualization process in PA-MSCs. Notably, several annotated genes typically upregulated during decidualization were downregulated in E2/P4-treated PA-MSCs, as shown in Supplementary Figure S5A. To explore the potential upstream regulators of this impairment, we examined the expression levels of ER and PR. Therefore, we revisited the expression levels of ER and PR. Surprisingly, we discovered that ER was more expressed in PA-MSCs than in normal MD-MSCs, as evidenced by flow cytometry analysis (Supplementary Figure S5B). This finding was further corroborated by immunohistochemical (IHC) staining performed on PAS and normal placenta biopsy samples (Supplementary Figure S5C). The severe FIGO grade 3 PAS (placenta percreta) is characterized by extensive disruption of the decidual membrane. Therefore, we selectively obtained biopsies exhibiting FIGO grade 2 PAS (placenta increta), which is distinguished by the presence of residual decidua, as observed in IHC staining (Figure 2D; Supplementary Figure S5C).

### Decidualization defects may not constitute the primary cause of dysregulation in trophoblast invasion, thereby leading to PAS

**FIGURE 3:**
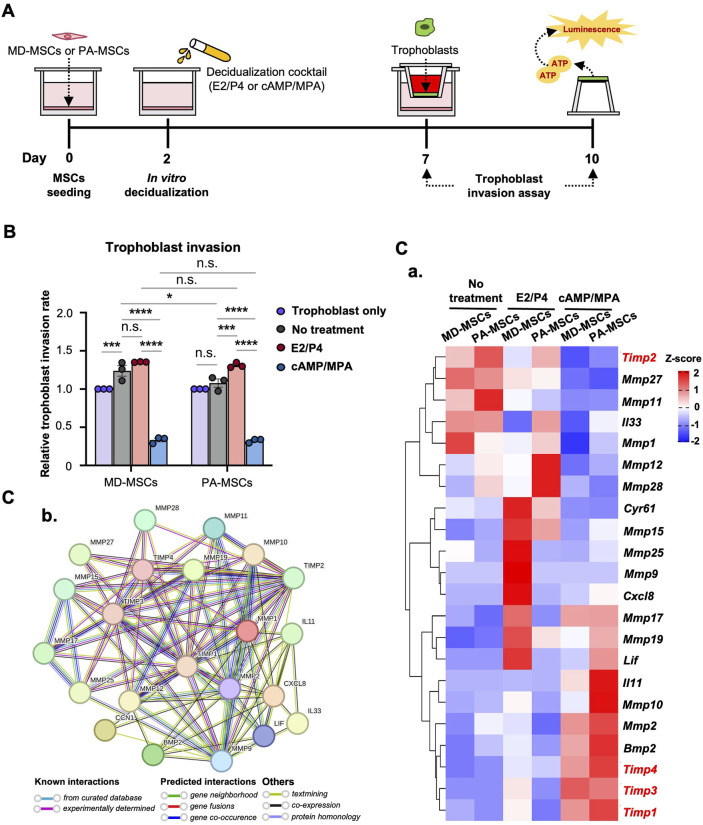
PA-MSCs and MD-MSCs have similar effects on trophoblast invasion through an MD-MSC-based co-culture system. **(A)** Schematic diagram of the co-culture system and timeline for the assessment of trophoblast invasion. **(B)** The effects of MD-MSCs and PA-MSCs under different decidualization treatments were compared to assess their impact on trophoblast invasion. Values are expressed as mean ± SEM. *p < 0.05, **p < 0.01, and ***p < 0.001, n.s.: not significant. **(C) (a)** The heatmap shows the potential molecules that are involved in the dynamic regulation of trophoblast invasion, annotated through the GEO database. Genes that negatively regulate trophoblast invasion were marked in red. Values are expressed as *z*-scores, which have been normalized to the gene expression levels. **(b)** Predicted networks of protein-protein interaction among annotated proteins through STRING analysis. The line between two nodes typically denotes the interaction between those two proteins, with the line’s colors reflecting the available resources of interactions.

Considering the association between PAS and dysregulated decidual cells impacting trophoblast invasion, we established a co-culture system to evaluate the interaction between PA-MSCs and trophoblasts. The trophoblast invasion timeline is depicted in Figure 3A. In this system, we made a surprising discovery, as illustrated in Figure 3B. We found that MD-MSCs treated with cAMP/MPA inhibited trophoblast invasion, while normal and E2/P4-treated MD-MSCs enhanced trophoblast invasion. This implies that MD-MSCs and De-MD-MSCs can dynamically regulate trophoblast invasion, aligning with the concept proposed in clinical research that “decidual cells act as a natural stop signal to inhibit excessive trophoblast invasion.” This phenomenon highlights the intricate interactions between decidual cells and trophoblasts and provides valuable insights into the regulation of trophoblast invasion during pregnancy.

E2/P4-treated De-PA-MSCs showed a statistically significant increase in the regulation of trophoblast invasion compared to the untreated group, as expected. In contrast, normal MD-MSCs did not show a significant difference in trophoblast invasion between E2/P4-treated and untreated conditions (Figure 3B). These results suggest that PA-MSCs may abnormally regulate trophoblast invasion under hormone decidualization. However, there was no significant difference in the regulation of trophoblast invasion between De-PA-MSCs and MD-MSCs under E2/P4 treatment. This finding challenges the assumption that impaired decidualization is one of the primary causes of PAS. Nevertheless, we investigated potential regulators and signaling pathways involved in trophoblast invasion regulation. The expression levels of 22 annotated genes associated with the regulation of trophoblast invasion are revealed through our RNA-seq database analysis. Notably, the genes negatively regulating trophoblast invasion, highlighted in red, were highly expressed in MD-MSCs under cAMP/MPA treatment (Figure 3C; Supplementary Data 2). Additionally, when comparing E2/P4-treated De-PA-MSCs to E2/P4-treated De-MD-MSCs, we observed lower expression levels of *Timp1*, *Timp3*, and *Timp4*, whereas *Timp2* was upregulated. *Timp2* inhibits active MMP-2 and helps activate pro-MMP-2 by forming a complex with MMP-2 and MMP-14 at the cell surface. Reduced TIMP-2 disrupts this balance, hindering trophoblast invasion (Bernardo and Fridman, 2003). In contrast, *Il33*, *Mmp12*, and *Mmp28* were significantly upregulated in the De-PA-MSCs under E2/P4 treatment (Figure 3C-a). The protein-protein interactions were analyzed by STRING for these proteins, encoded by these genes, and exhibited a high density of interactions involved in regulating trophoblast invasion. Particularly, tissue inhibitors of metalloproteinases (TIMPs) and matrix metalloproteinases (MMPs) exhibit a high level of evidence of known interactions (Figure 3C-b). These analyses suggest that the innate regulatory mechanisms governing trophoblast invasion through E2/P4 decidualization in PA-MSCs differ from those observed in normal MD-MSCs. However, the differential expressions of these genes were not sufficient to account for the abnormal trophoblast invasion. Further research is necessary to fully understand the underlying mechanisms contributing to PAS. Particular attention should be given to dNK cells due to their pivotal role in pregnancy through their intrinsic interactions with decidual cells and trophoblasts within the physiological microenvironment.

### The induction of dNK-like cells from pNK cells is potentially achievable through an MD-MSC-based co-culture system

**FIGURE 4:**
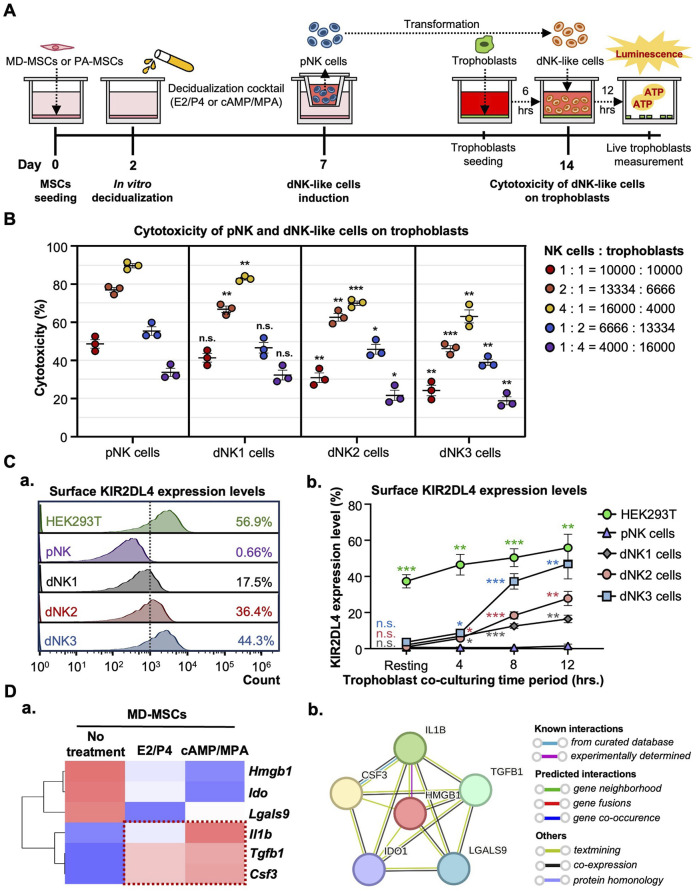
MD-MSCs can potentially convert dNK-like cells from pNK cells. **(A)** A schematic diagram and timeline depict the assessment of immune suppression in dNK-like cells using a co-culture system. **(B)** Evaluation of cytotoxicity involves three different MD-MSC-induced dNK-like cells interacting with trophoblasts. **(C) (a)** Flow cytometry analysis and **(b)** quantitative statistics represent surface KIR2DL4 expression levels on different subtypes of NK cells after co-culture with trophoblasts for 12 h. HEK293T serves as the positive control for KIR2DL4. Values are normalized to the IgG control and expressed as mean ± SEM. Significance levels are denoted as *p < 0.05, **p < 0.01, and ***p < 0.001, with all comparisons made against the pNK cell group. **(D) (a)** The heatmap illustrates three potential secreted proteins encoding genes that regulate the conversion of pNK cells into dNK-like cells. Values are expressed as z-scores, which have been normalized to the gene expression levels. **(b)** Predicted networks of protein-protein interaction among annotated proteins through STRING analysis. The line between two nodes typically represents the interaction between those two proteins, with the colors of the line reflecting the available resources for interactions. pNK cell: peripheral NK cells; dNK1 cells: MD-MSC-induced dNK-like cells; dNK2 cells: E2/P4-treated De-MD-MSC-induced dNK-like cells; dNK3 cells: cAMP/MPA-treated De-MD-MSC-induced dNK-like cells.

It is well-established that, apart from decidual cells, dNK cells also play a crucial role in blastocyst implantation and placental formation. Due to limitations in acquiring dNK cells, we attempt to facilitate the *in vitro* conversion of dNK cells from pNK cells through co-culturing with MD-MSCs or De-MD-MSCs. The primary indicator of the successful conversion of dNK cells is the reduced cytotoxicity characteristic of dNK cells. The timeline of the dNK-like cell conversion and the assessments are depicted in Figure 4A. As the results showed, after co-culturing with MD-MSCs or De-MD-MSCs, the cytotoxicity of NK cells on trophoblasts decreased at each ratio of effector cells and target cells compared with pNK cells (Figure 4B). Particularly in the dNK cell batches, dNK2 and dNK3 cells, the cytolytic ability was dramatically reduced by about 20%–30% compared with pNK cells at the cell number ratio of 4:1 (Figure 4B).

To exclude the possibility that the decline in NK cell cytotoxicity is not attributable to functional loss post-co-culture MD-MSCs, we further investigated the upregulated expression levels of KIR2DL4, specifically expressed on dNK cell surfaces, to bind with HLA-G on trophoblast surfaces and activate the inhibitory signal contributing to immunotolerance to prevent trophoblasts from being cytolyzed. Firstly, we measured the levels of HLA-G on trophoblasts to confirm its presence (Supplementary Figure S7A). As anticipated, KIR2DL4 expression was notably higher, particularly on dNK cell batches, dNK2 and dNK3 cells, with percentages reaching 36.4% and 44.3% positive compared to the IgG control (Figure 4C-a). The quantified statistics, normalized with pNK cells, demonstrated a dramatic increase in the expression levels of KIR2DL4 after co-culturing with trophoblasts (Figure 4C-b; Supplementary Figure S7B). The upregulated expression level of KIR2DL4 was consistent with the reduced cytotoxic ability. Apart from the inhibitory receptor expression, the cytokines and chemokines associated with immunotolerance secreted by decidual cells are found in the RNA-seq database, including *Csf3*, *Il1β*, and *Tgfb1* (Figure 4D-a; Supplementary Data 3). The protein-protein interactions analyzed by STRING for these six secreted proteins exhibit interactions in regulating immune tolerance for dNK cells. Of noteworthy significance is the interaction between IL1β and CSF3, which is strongly supported by evidence from curated databases (Figure 4D-b). These results suggest that MD-MSCs can potentially convert pNK cells into dNK-like cells and potentially mimic the immunotolerance on trophoblasts *in vitro*. Moreover, we characterized dNK2 cells and compared them to pNK cells using CD56 and CD9 markers (Albini and Noonan, 2021; Zhang et al., 2024). As shown in Supplementary Figure S8, dNK2 cells exhibit a slight differentiation tendency, marked by CD56^bright^CD9^high^ expression, in contrast to pNK cells.

### Identification of highly proliferated dNK-like cells in PA-MSCs

**FIGURE 5:**
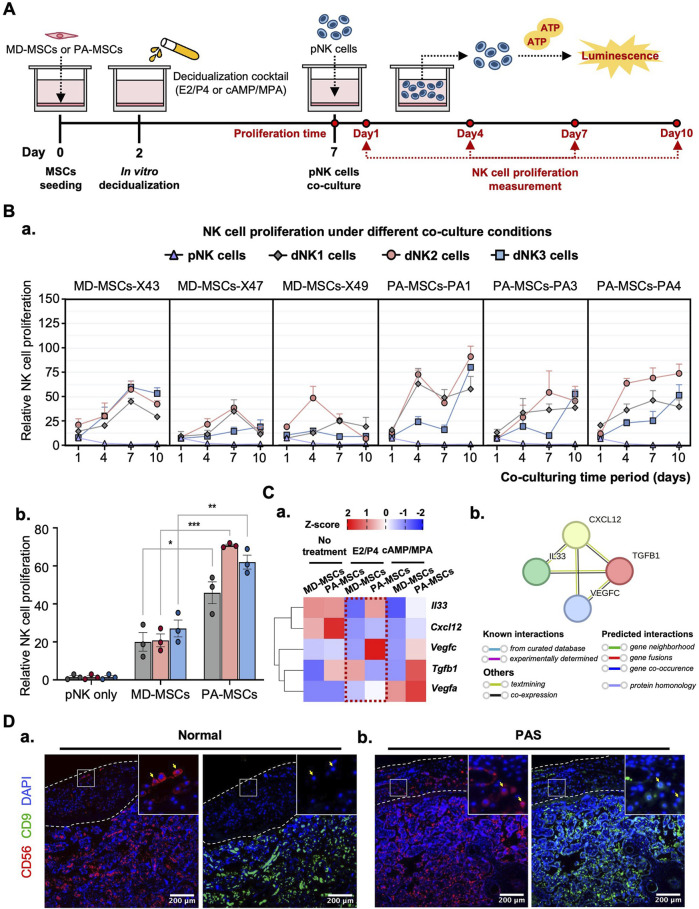
PA-MSCs demonstrate a greater capacity to enhance dNK-like cell proliferation than normal MD-MSCs. **(A)** A schematic diagram of the co-culture system for the dNK-like cell proliferation assay using MD-MSCs and PA-MSCs. **(B) (a)** The proliferation of dNK-like cells was measured at specific time points: 1, 4, 7, and 10 days under various conditions, including co-culture with MD-MSCs, PA-MSCs, and their corresponding decidual counterparts. **(b)** The effects of MD-MSCs, PA-MSCs, and their decidual counterparts on dNK-like cell proliferation were quantified at day 10. Values are expressed as mean ± SEM. *p < 0.05, **p < 0.01, and ***p < 0.001. **(C) (a)** The heat map illustrates the expression patterns of annotated secreted proteins responsible for stimulating dNK-like cell proliferation. Values are expressed as z-scores, which have been normalized to the gene expression levels. **(b)** Predicted networks of protein-protein interaction among annotated proteins through STRING analysis. The line between two nodes typically denotes the interaction between those two proteins, with the colors of the line reflecting the available resources of interactions. The thickness of the line is about the strength of the interactions. **(D)** Immunofluorescence staining of dNK cells in **(a)** a normal placenta and **(b)** a PAS placenta. The white-lined sections represent the decidual membrane. dNK cells are stained with CD56 (red) and CD9 (green). pNK cell: peripheral NK cells; dNK1 cells: MD-MSC-induced dNK-like cells; dNK2 cells: E2/P4-treated De-MD-MSC-induced dNK-like cells; dNK3 cells: cAMP/MPA-treated De-MD-MSC-induced dNK-like cells.

Following the successful induction of dNK-like cells *in vitro*, we conducted a further investigation to examine the differential effects of MD-MSCs and PA-MSCs on dNK-like cells in an attempt to find the potential causes of PAS. Initially, we assessed NK cell proliferation under co-culture conditions with MD-MSCs or PA-MSCs following various decidualization inductions (Figure 5A). The experimental timeline spanned 10 days, with day 7 corresponding to the dNK-like cell conversion period. In the NK cell proliferation assay, we made an intriguing observation: PA-MSCs demonstrated a greater capacity to enhance NK cell proliferation compared to normal MD-MSCs, particularly in the E2/P4-treated groups (Figure 5B-a). The quantified analysis clearly showed a three-fold increase in NK cell proliferation after co-culture with E2/P4-treated PA-MSCs compared to E2/P4-treated MD-MSCs on day 10 (Figure 5B-b). This observation prompted us to explore the relationship between NK cell proliferation and trophoblast invasion further. Additionally, our RNA-seq analysis identified cytokines, known as key regulators of NK cell proliferation, which were predominantly upregulated in the PA-MSCs groups. Significantly, five genes potentially enhancing NK cell proliferation in E2/P4-treated De-PA-MSCs were annotated, including *Cxcl12*, *Il33, Vegfa*, *Vegfc,* and *Tgfb1* (Figure 5C-a; Supplementary Data 4), with the predicted interactions of these secreted proteins shown in Figure 5C-b. VEGFA was not displayed in the interaction panel due to the absence of information in the STRING database. These findings suggested a potential association between abnormal NK cell proliferation and the development of PAS. Moreover, the increased proliferation of dNK cells in the placenta of PAS patients is also evident through immunofluorescence staining for CD56 and CD9 markers (Figure 5D). Consequently, we propose the hypothesis that Abnormal dNK cell proliferation may represent a significant contributing factor to the pathogenesis of PAS.

### Increased dNK cell numbers may represent one of the key factors contributing to PAS

**FIGURE 6:**
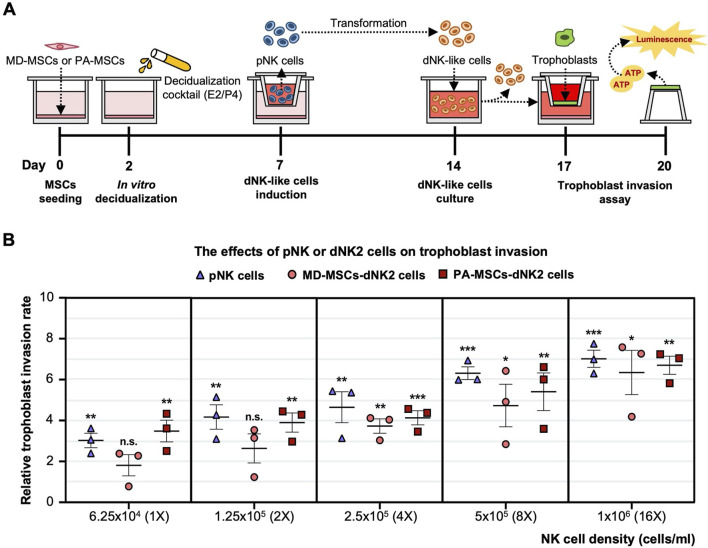
Enhanced trophoblast invasion was observed under E2/P4 treated PA-induced dNK-like cells with a dose-dependent correlation. **(A)** A schematic diagram and timeline depict the assessment of trophoblast invasion under MD-MSC or PA-induced dNK2 cell-derived conditioned medium. **(B)** Dose-dependent correlation in three subtypes of NK cells. Values are expressed as mean ± SEM. Significance levels are denoted as *p < 0.05, **p < 0.01, and ***p < 0.001, with all comparisons made against the pNK cell group. pNK cell: peripheral NK cells; dNK2 cells: E2/P4-treated De-MD-MSC-induced dNK-like cells.

**FIGURE 7:**
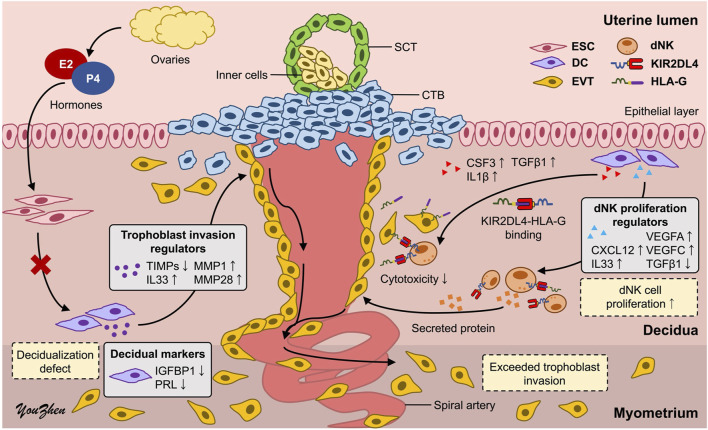
Schematic representation of the underlying mechanisms of PAS. The high proliferation of dNK cells induced by defective decidual cells may dysregulate trophoblast invasion, leading to PAS. CTB: cytotrophoblast; SCT: syncytiotrophoblast; ESC: endometrial stromal cell; DC: decidual cell; EVT: extravillous trophoblast; dNK: decidual natural killer cell.

To assess the earlier presumption that PAS could be attributed to dysregulated trophoblast invasion, we modified the previously established co-culture system to investigate the distinct impact of increasing the dNK cell batch dNK2 cell numbers on trophoblast invasion. The experimental timeline for this study is depicted in Figure 6A. Given the complex interplay among the cells involved, we simplified the co-culture system by utilizing a dNK2 cell-conditioned medium. We anticipated that dNK2 cells would exhibit an enhanced capacity to promote trophoblast invasion with a dose-dependent correlation (Figure 6B). Furthermore, dNK2 cells induced by PA-MSCs demonstrated a greater capacity to enhance trophoblast invasion compared to those induced by MD-MSCs (Figure 6B). Statistical analysis was performed by normalizing the results with the control trophoblast alone group. This outcome indirectly suggests that increased dNK cell proliferation may be a pivotal factor in abnormal trophoblast invasion, potentially contributing to PAS as depicted in Figure 7. The novel finding sheds light on the mechanisms underlying PAS and lays the groundwork for the development of treatments for this condition.

## Discussion

Previous studies have revealed the critical role that moderate trophoblast invasion plays in a successful pregnancy. Dysregulation of trophoblast invasion can lead to maternal lethal diseases, like the most severe pattern, PAS. PAS is associated with complicated interactions among decidual cells, trophoblasts, and dNK cells. The potential mechanisms of the PAS have not been clarified due to the limitations of the cell derivation.

In the published studies, primary ESCs and even decidual cells were predominantly obtained from menstrual blood (Sanche et al., 2021; Bozorgmehr et al., 2020) or endometrial biopsies, including curettage (Wu et al., 2017; Pei et al., 2019) and elective termination of pregnancy samples (Menkhorst et al., 2023; Lindau et al., 2021). However, these sources of ESCs have limitations regarding cell number and purity and involve invasive surgical procedures that may cause discomfort to the subjects. Furthermore, obtaining specimens from normal women has proven particularly challenging due to ethical concerns. In this study, we have identified a distinct cell type among ESCs from normal placenta characterized by its potential for decidualization and high capacity for *in vitro* amplification. Biomarker investigations validated the decidualization process of MD-MSCs, with particular attention to recognized decidual cell markers such as *Prl* and *Igfbp1* (Telgmann and Gellersen, 1998; Diniz-da-Costa et al., 2021). Our study observed a unique upregulation of *Scara5* (Lucas et al., 2020) in De-MD-MSCs, which has been suggested to play a pivotal role in crucial cellular functions during decidualization. Studies indicate that SCARA5 is downregulated in senescent decidual cells, suggesting its potential therapeutic implications in reproductive disorders (Rawlings et al., 2021; Hou et al., 2023; Szwarc et al., 2018). Further research is warranted to fully elucidate the mechanisms underlying the involvement of SCARA5 in decidualization and its implications for reproductive health. Given their unique *in vitro* decidualization potential and their ability to circumvent ethical issues, MD-MSCs stand out as a potentially valuable model for advancing the study of placental development and associated diseases.

In the trophoblast invasion assay, the validated EVT cell line 3A-subE was used in this study. We found that MD-MSCs treated with cAMP/MPA negatively regulate trophoblast invasion, in contrast to E2/P4-treated MD-MSCs, which enhance trophoblast invasion. The regulation of trophoblast invasion involves intricate cell-cell interactions and activation of multiple signaling pathways. Proteins secreted from decidual cells can either promote or inhibit trophoblast invasion, thus modulating trophoblast invasion levels. Gene expression analysis suggests that genes associated with trophoblast invasion enhancement (*Mmps*) are reduced, while genes associated with inhibiting trophoblast invasion (*Timps*) are upregulated in cAMP/MPA-treated MD-MSCs, consistent with their inhibitory effect on trophoblasts (Sharma et al., 2016; Zhu et al., 2012; Hamutoğlu et al., 2020; Li et al., 2021; Haider et al., 2022). In contrast, E2/P4 treatment sustains or increases *Mmp* expression relative to *Timps*, creating an environment that promotes trophoblast invasion. Of particular interest is the upregulation of *Timp2*, which uniquely contributes to both the inhibition and activation of MMP2, reflecting a finely balanced regulatory mechanism controlling matrix remodeling. These findings underscore how distinct hormonal signals differentially modulate the MMP/TIMP axis to influence trophoblast function and decidual remodeling.

Given that we observed that the decidual membrane was not absent in PAS patients, this finding contradicts the hallmark characteristic of PAS: the absence of the decidual membrane. Beyond the loss of the decidual membrane, poor decidualization and dysfunctional decidual cells may also play critical roles in the development of PAS (Afshar et al., 2024; Morlando and Collins, 2020; Arak et al., 2023). Consequently, we sought to further investigate the underlying mechanisms by utilizing pathological MD-MSCs, focusing on the remnants of the residual decidual membrane. We observed diminished morphological changes and decreased expression levels of PRL and IGFBP1, both at mRNA and protein levels, *in vitro* and *in vivo*. Additionally, besides these two decidual markers, other annotated upregulated genes under decidualization were found to be downregulated under E2/P4 treatment. This observation may be due to abnormally high ER expression, which has been associated with decidualization defects and endometriosis (Chantalat et al., 2020; Han et al., 2019). Elevated ERβ expression and activity in endometriosis promote lesion growth by inhibiting apoptosis, activating inflammatory pathways, defective decidualization, and enhancing invasion through epithelial–mesenchymal transition (Han et al., 2015). Regarding trophoblast invasion, no discernible effects on trophoblast invasion were observed between De-MD-MSCs and De-PA-MSCs. However, RNA-seq data revealed differential expression of molecules involved in trophoblast invasion regulation in E2/P4 treated De-PA-MSCs, notably downregulated *Timp1*, *Timp3* and *Timp4* (Szwarc et al., 2018), (Afshar et al., 2024; Morlando and Collins, 2020), (Tapia-Pizarro et al., 2013; Ma et al., 2014) and upregulated *Il33* (Chen et al., 2018; Valero-Pacheco et al., 2022), *Mmp1*, *Mmp12* and *Mmp28* (Deng et al., 2015; Li et al., 2003; Arora et al., 2023), which may promote trophoblast invasion. Based on these results, we propose two possible explanations. Firstly, it is conceivable that the drug potency of cAMP/MPA is too high to reveal the decidualization defect in PA-MSCs. Secondly, abnormal trophoblast invasion may not be directly attributed to the inherent properties of defective decidual cells.

To investigate the indirect effects on trophoblast invasion, dNK cells were included in the co-culture system to explore their interactions with decidual cells and trophoblasts. At the maternal-fetal interface, dNK cells play a crucial role in promoting immune tolerance through specific interactions between KIRs on dNK cells and HLA expressed on trophoblasts (Bhattacharya et al., 2015). Notably, KIR2DL4 on maternal dNK cells can interact with soluble HLA-G (sHLA-G) from fetal trophoblast cells, potentially modulating immune responses and promoting tolerance to the semi-allogeneic fetus during pregnancy (Hanna et al., 2006; Velicky et al., 2016). This interaction is pivotal in regulating maternal immune responses and maintaining the integrity of the maternal-fetal interface. However, due to the challenge in deriving dNK cells, we opted for *in vitro* induction methods to address this issue. Instead of chemical induction using TGFβ and demethylation agents (Cerdeira et al., 2013), we employed decidual cell induction methods to closely mimic the physiological microenvironment (Vacca et al., 2011). Interestingly, De-MD-MSCs demonstrated the ability to induce dNK-like cells from pNK cells. Although these dNK-like cells did not exhibit markedly high expression of dNK cell surface markers, characterized by CD56^bright^CD16^−^CD9^bright^CD49a^bright^, their induction could potentially be enhanced under hypoxic conditions (Du et al., 2022). Despite the limited expression of surface markers, KIR2DL4 expression was significantly upregulated in dNK-like cells when co-cultured with De-MD-MSCs treated with either E2/P4 or cAMP/MPA. The observed reduction in immune response may be attributed to cytokines secreted by decidual cells, including *Csf3*, *Il-1β,* and *Tgfb1* (Dwyer et al., 2022; Wen et al., 2023), as suggested by RNA-seq data. Furthermore, we made a novel observation regarding the abnormal interaction between PA-MSCs and dNK-like cells. PA-MSCs exhibited a significant capacity to enhance dNK-like cell proliferation, as evidenced by the increased dNK cells in PAS placenta from immunofluorescence images. However, the underlying mechanisms driving this abnormal proliferation of dNK cells in PAS have yet to be fully elucidated. Only a few published studies proposed that the reduced proliferation of dNK cells may be one of the contributing factors to PAS (Khairy et al., 2017). Thus, further evidence is required to clarify the correlation between dNK cell proliferation and PAS.

Based on the observed decidualization defect and the highly proliferated dNK-like cells induced by PA-MSCs, several secreted proteins implicated in promoting dNK cell proliferation and correlating with trophoblast invasion are found to be upregulated and annotated in RNA-seq data from PA-MSCs compared to MD-MSCs. CXCL12 secreted by decidual cells can bind to CXCR4 receptors on dNK cells or trophoblasts, indirectly or directly inducing trophoblast differentiation, invasion, and spiral artery remodeling, which may be through activating downstream PI3K-Akt and MAPK-ERK signaling pathways (Supplementary Figure S6; Supplementary Data 5) (Zhu et al., 2012; Arck and Hecher, 2013; Wang et al., 2015; Li et al., 2024). In addition, IL33 has been found to activate the NF-κB and MAPK pathways and is essential for regulating trophoblast invasion and placental development by modulating the immune environment (Supplementary Figure S6) (Chen et al., 2018; Valero-Pacheco et al., 2022). Notably, TGFβ1, which has been suggested in evidence to suppress NK cell proliferation via SMAD-dependent signaling (Yang et al., 2021; Lamb et al., 2021), is downregulated in E2/P4-treated De-PA-MSCs, suggesting a reduction in immunoregulatory control that may permit excessive trophoblast activity. Interestingly, the TGF-β signaling pathway is enriched at the transcriptomic level, likely driven by upregulation of other pathway components, which may reflect compensatory or context-specific activation rather than direct TGFβ1-mediated signaling (Supplementary Figure S6). Furthermore, VEGFA and VEGFC, secreted by both decidual cells and dNK cells, not only enhance trophoblast invasion but also promote angiogenesis and spiral artery remodeling, potentially leading to significant bleeding in PAS (El-Badawy et al., 2023; Rekowska et al., 2023). In our study, the upregulated and downregulated genes suggest a positive correlation among dNK cell proliferation, trophoblast invasion, and spiral artery remodeling. Functional validation reveals that conditioned medium derived from dNK-cells induced by E2/P4-treated De-PA-MSCs has a greater capacity to enhance trophoblast invasion in a dose-dependent manner compared to dNK-like cells induced by De-MD-MSCs.

These findings provide new insights into the underlying mechanisms of PAS through the decidualization defect and subsequent abnormal interactions among decidual, dNK, and trophoblast cells. MD-MSCs have the potential to break the limitations of *in vitro* studies related to complex placental diseases and may significantly contribute to advancements in human health. However, the MD-MSCs-based co-culture model for studying placental disorders still presents challenges that require further efforts to overcome. The physiological identity of MD-MSCs in the endometrium requires more evidence to substantiate their correlation with decidualization and their role in placental formation, thereby highlighting the importance of this cell type. Due to a small clinical sample size, our results need further validation to ensure consistent findings through both *in vitro* cell assays and *in vivo* mouse models. The MD-MSC-based co-culture system needs to be augmented by co-culturing with various cell types to better mimic the complex microenvironment. Additionally, single-cell RNA-seq and cytokine array analyses should be conducted to validate the aforementioned results. Regarding trophoblast behavior, while this study focused on invasion capacity, the expression of differentiation markers also plays a critical role in regulating trophoblast function and warrants further investigation. We anticipate identifying specific molecular pathways to comprehensively elucidate the mechanisms underlying complex PAS in future research endeavors.

## Conclusion

This study highlights the development of an MD-MSC-based system for *in vitro* investigation of the intricate interactions among decidual, dNK, and trophoblast cell induction and their implications for PAS. Utilizing the MD-MSC-based co-culture system, decidualization defects characterized by decreased morphological changes and expression of decidual markers were observed in the residual decidual membrane of PA-MSCs. Additionally, MD-MSCs demonstrated the capacity to induce the differentiation of pNK cells into dNK-like cells, as evidenced by reduced cytotoxicity on trophoblasts and increased expression of KIR2DL4. Furthermore, PA-MSCs exhibited a high capacity to induce dNK-like cell proliferation, indirectly enhancing trophoblast invasion and spiral artery remodeling, possibly through CXCL12, TGFβ1, VEGFA, and VEGFC secretion. These findings provide new insights and lay the groundwork for further elucidating the complex pathophysiology of PAS through the novel MD-MSC cell type.

## Data Availability

The original contributions presented in the study are included in the article and/or Supplementary Material, further inquiries can be directed to the corresponding authors.
