# Correction: Dysregulation of decidual NK cell proliferation by impaired decidual cells: a potential contributor to excessive trophoblast invasion in placenta accreta spectrum

**DOI:** 10.3389/fcell.2025.1696297

**Published:** 2025-11-11

**Authors:** You-Zhen Liu, Hsin-Hung Lin, Meng-Shiue Wu, Jin-Chung Shih, Thai-Yen Ling

**Affiliations:** 1 Graduate Institute of Pharmacology, National Taiwan University College of Medicine, Taipei, Taiwan; 2 MediDiamond Inc., Taipei, Taiwan; 3 LuminX Biotech Co., Ltd., Taipei, Taiwan; 4 Department of Obstetrics and Gynecology, National Taiwan University Hospital, Taipei, Taiwan

**Keywords:** decidualization, placenta accreta spectrum (PAS), trophoblast invasion, decidual natural killer cell, immune tolerace

Affiliation ^1^Graduate Institute of Pharmacology, National Taiwan University College of Medicine, Taipei, Taiwan was erroneously given as ^1^Graduate Institute of Pharmacology, National Taiwan University College of Medicine, Taipei, Taiwan, United States.

The original article has been updated.

