# Supplementary material for: Dysregulation of decidual NK cell proliferation by impaired decidual cells: a potential contributor to excessive trophoblast invasion in placenta accreta spectrum
Source: Front Cell Dev Biol. 2025 Jul 21;13:1618461. doi: 10.3389/fcell.2025.1618461 (PMC12320386; doi:10.3389/fcell.2025.1618461)
Supplement: Supplementary file 1 [file DataSheet1.docx]

**Dysregulation of Decidual NK Cell Proliferation by Impaired Decidual Cells: A Potential Contributor to Excessive Trophoblast Invasion in Placenta Accreta Spectrum**

**Authors**

You-Zhen Liu^1†^, Jin-Chung Shih^2†^, Meng-Shiue Wu^3^, Hsin-Hung Lin^1,3,4,5*^, Thai-Yen Ling^1*^

**Institutions**

^1^Graduate Institute of Pharmacology, National Taiwan University College of Medicine, Taiwan

^2^Department of Obstetrics and Gynecology, National Taiwan University Hospital, Taipei, Taiwan

^3^VitaSpring Biomedical Co. Ltd., USA

^4^MediDiamond Inc., Taiwan

^5^LuminX Biotech Co. Ltd., Taiwan

**Corresponding Author:**

^†^ These authors contributed equally to this work.

* Corresponding author:

Hsin-Hung Lin

Chief Technology Officer, MediDiamond Inc.

Tel: 886-2-26558597; Email: [HHLin@medidiamondinc.com](mailto:HHLin@medidiamondinc.com)

Thai-Yen Ling

Associate Professor & Director, Institute of Pharmacology, College of Medicine, National Taiwan University, Taiwan

Tel: 886-2-23123456 ext. 288322; Email: [tyling@ntu.edu.tw](mailto:tyling@ntu.edu.tw)

**Supplementary figures**

**A**

**
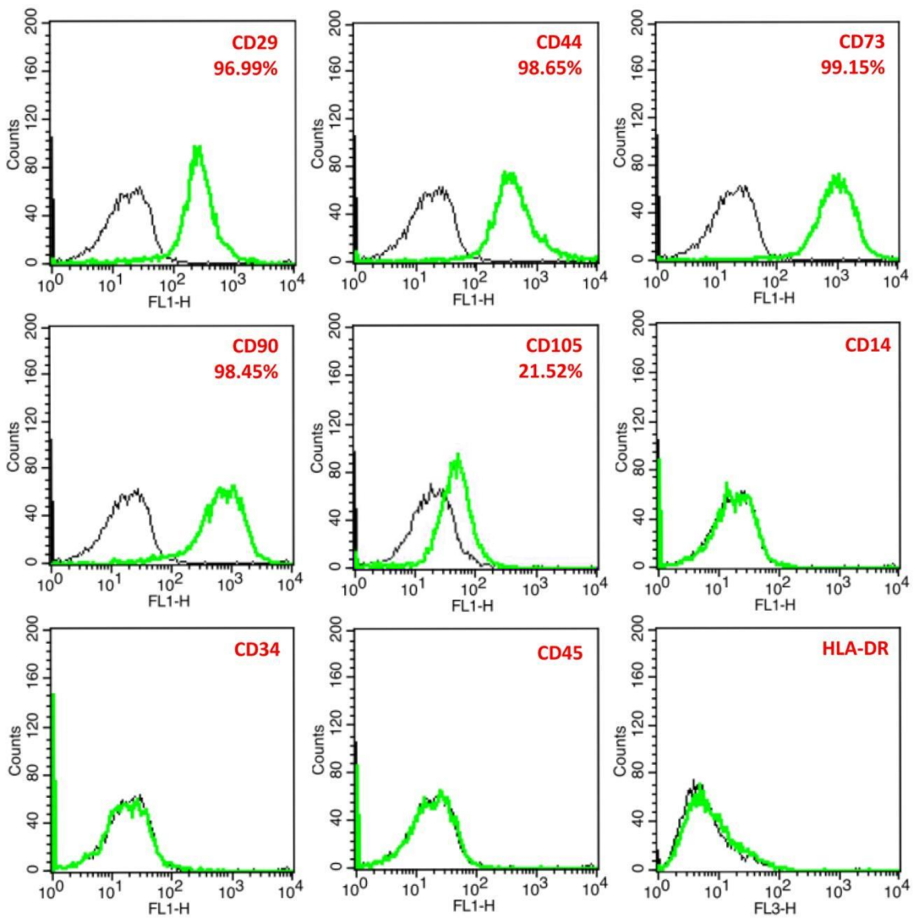
**

**B**

**
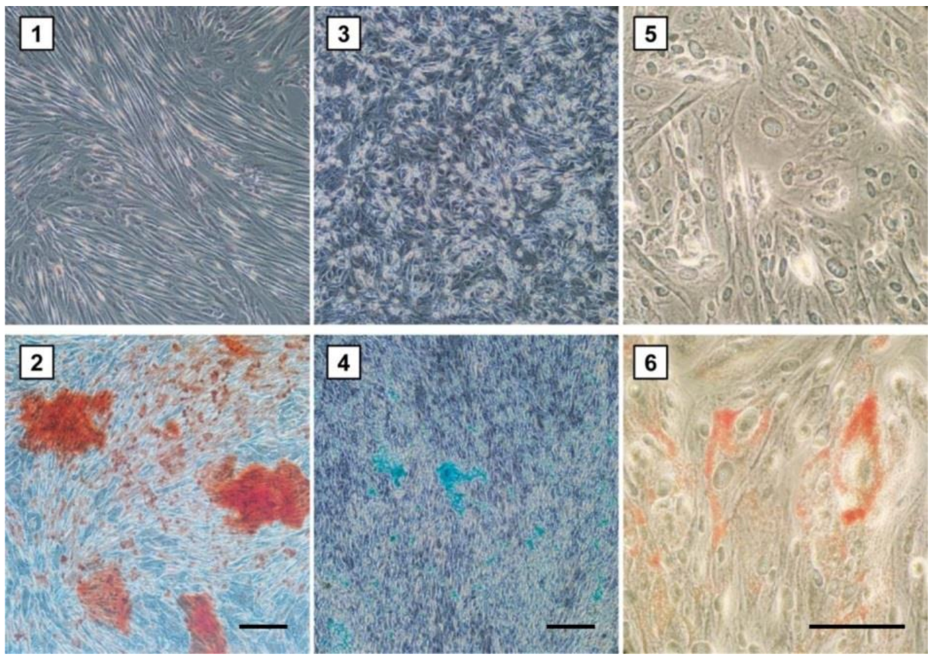
**

**Supplementary Figure 1 ⏐ Characterization of MD-MSCs through immunophenotype analysis and differentiation potential assessment (Su et al., 2017).**

**(A)** Immunophenotypic analysis of MD-MSCs reveals the expression of specific markers, including CD29+, CD44+, CD73+, CD90+, CD105+, CD14–, CD34–, CD45–, and HLA-DR–. Control groups are represented in black, and experimental groups are shown in green. **(B)** *In vitro* differentiation potential of MD-MSCs into osteogenic (1, 2), chondrogenic (3, 4), and adipogenic (5, 6) lineages. Panels (1), (3), and (5) display results from the corresponding control groups. Scale bar: 200 μm.


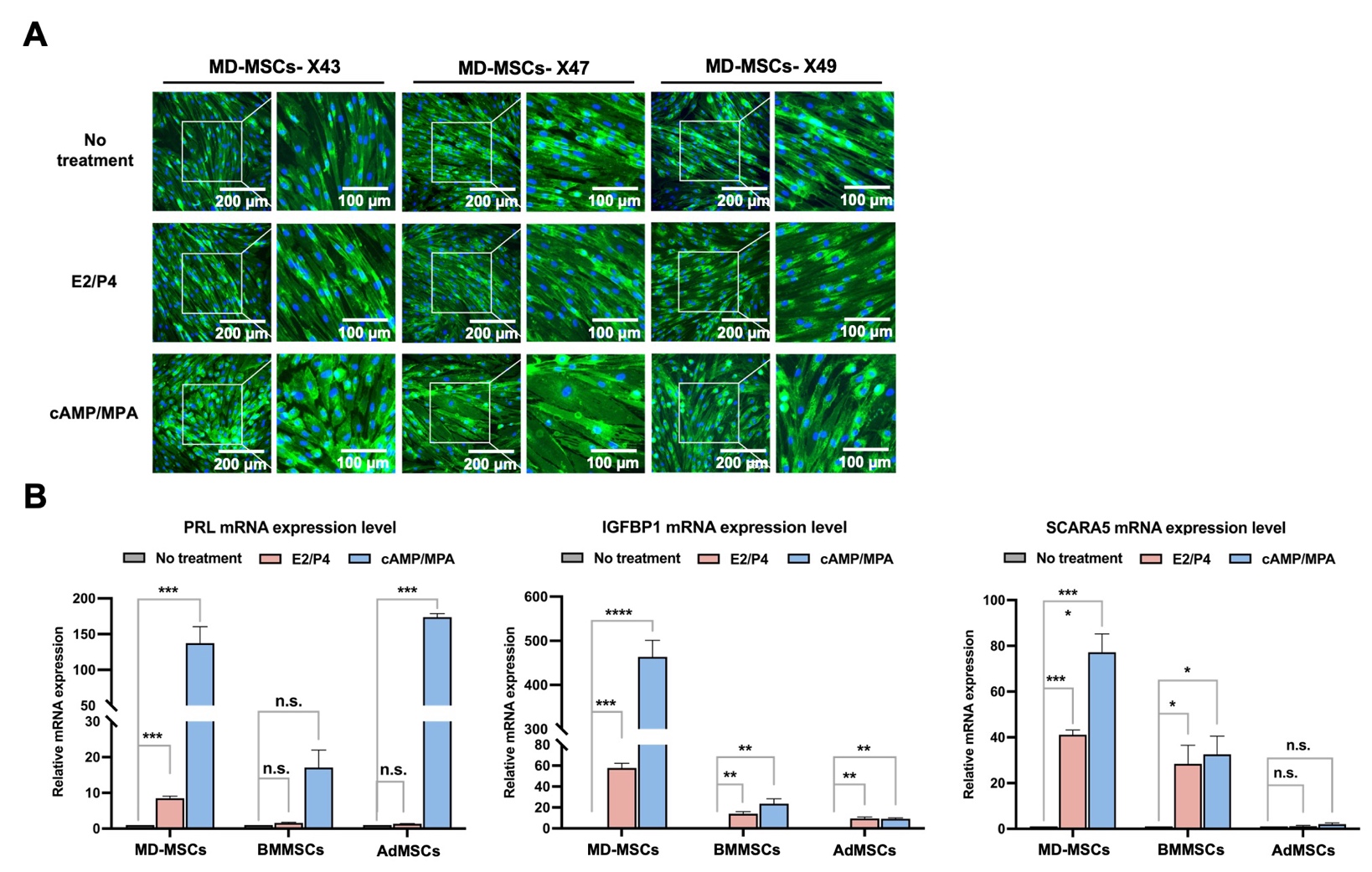


**Supplementary Figure 2 ⏐ Characterization of MD-MSCs for decidualization potential.**

**(A)** (a) Representative images and (b) quantified analysis of triplicate biological repeats across three batches of MD-MSCs for evaluating morphological changes under decidualization. **(B)** Three decidual cell markers, including *Prl*, *Igfbp1*, and *Scara5*, exhibit organ-specific expression through E2/P4 decidualization induction. Values are expressed as mean ± SEM. *p < 0.05, **p < 0.01, ***p < 0.001.


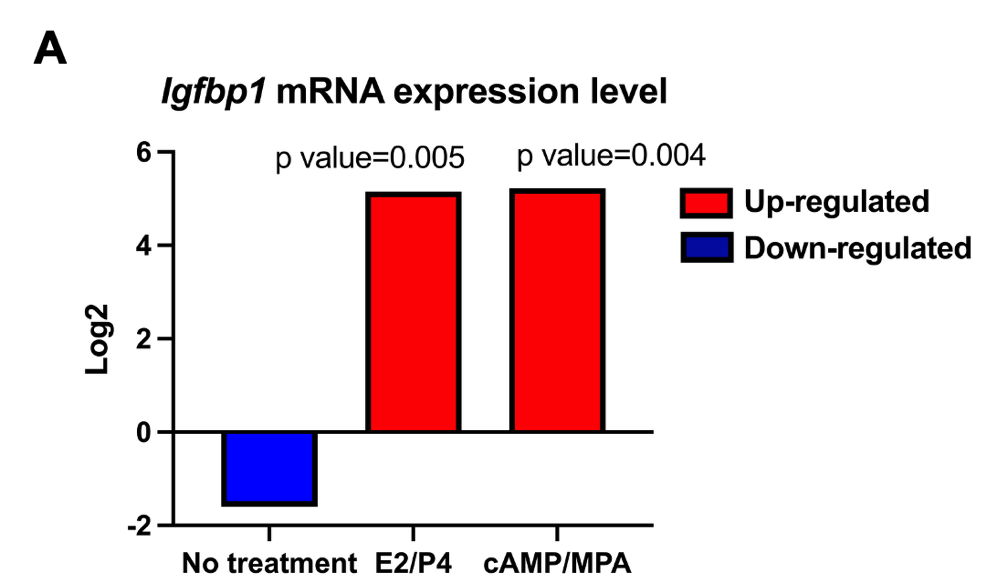


**Supplementary Figure 3 ⏐ *Igfbp1* gene expression level from RNA-seq database.**

**(A)** mRNA expression levels of *Igfbp1* under various decidualization treatments.

**
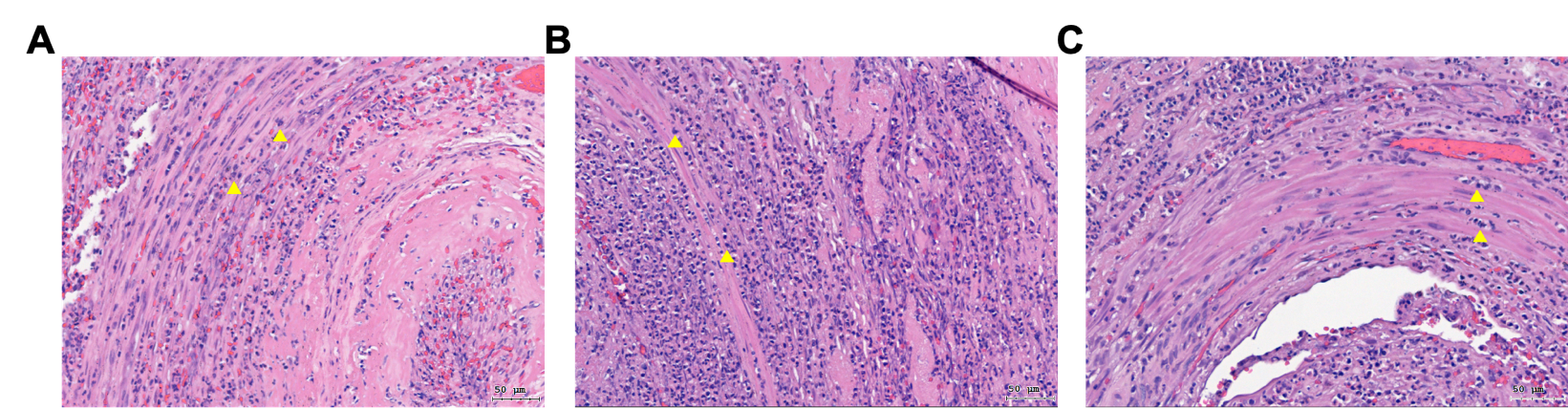
**

**Supplementary Figure 4 ⏐ Histopathological Analysis of PA-MSCs**

**(A)** iCTB invasion into the myometrium in PA-MSC-1, as shown by H&E staining. **(B)** H&E staining of PA-MSC-3 revealing the extent of trophoblast penetration into the myometrial tissue. **(C)** In PA-MSC-4, trophoblast cells are observed infiltrating the myometrium, as indicated by H&E staining. Yellow arrows indicate iCTBs. Scale bar: 50 µm.

**
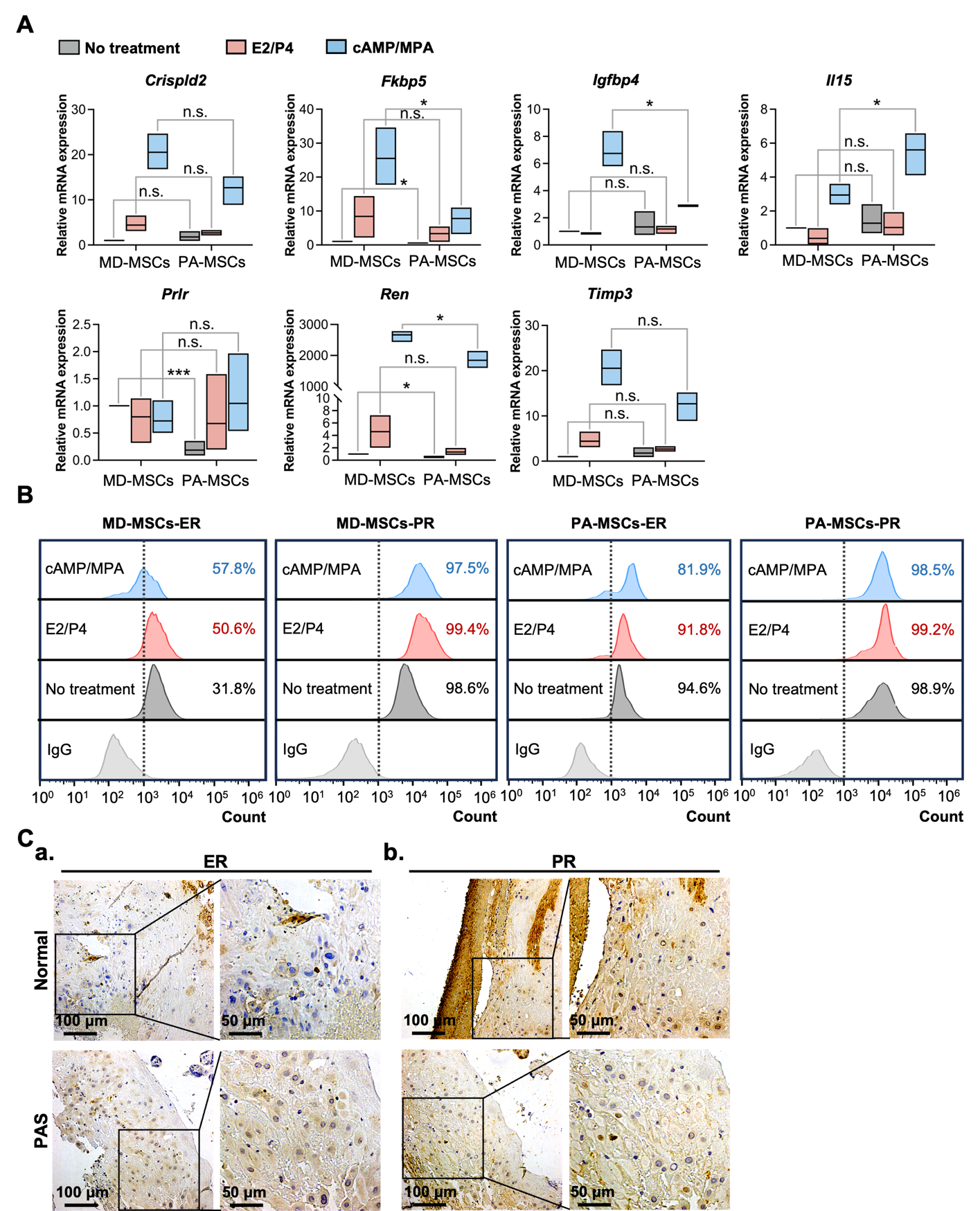
**

**Supplementary Figure 5 ⏐ Characterization of PA-MSCs for decidualization defects.**

**(A)** Annotated up-regulated genes are validated with RT-PCR analysis under different decidualization inductions in PA-MSCs compared with MD-MSCs. Values are expressed as mean ± SEM. *p < 0.05, **p < 0.01, ***p < 0.001. **(B)** ER and PR protein expression levels under decidualization treatments in MD-MSCs and PA-MSCs. Values are normalized to the IgG control. **(C)** IHC staining images demonstrate the ER and PR expression levels in normal and pathological placenta biopsies from PA-MSCs patients.

**Supplementary Figure 6 ⏐ KEGG pathway enrichment analysis of differentially expressed genes between PA-MSCs and MD-MSCs under E2/P4 treatment.**

**(A)** KEGG pathway enrichment analysis of upregulated and downregulated genes in PA-MSCs compared to MD-MSCs under E2/P4 treatment. **(B)** Detailed KEGG pathway enrichment analysis of upregulated genes in PA-MSCs compared to MD-MSCs under E2/P4 treatment. The PI3K-Akt and MAPK signaling pathways are highlighted in the panel.


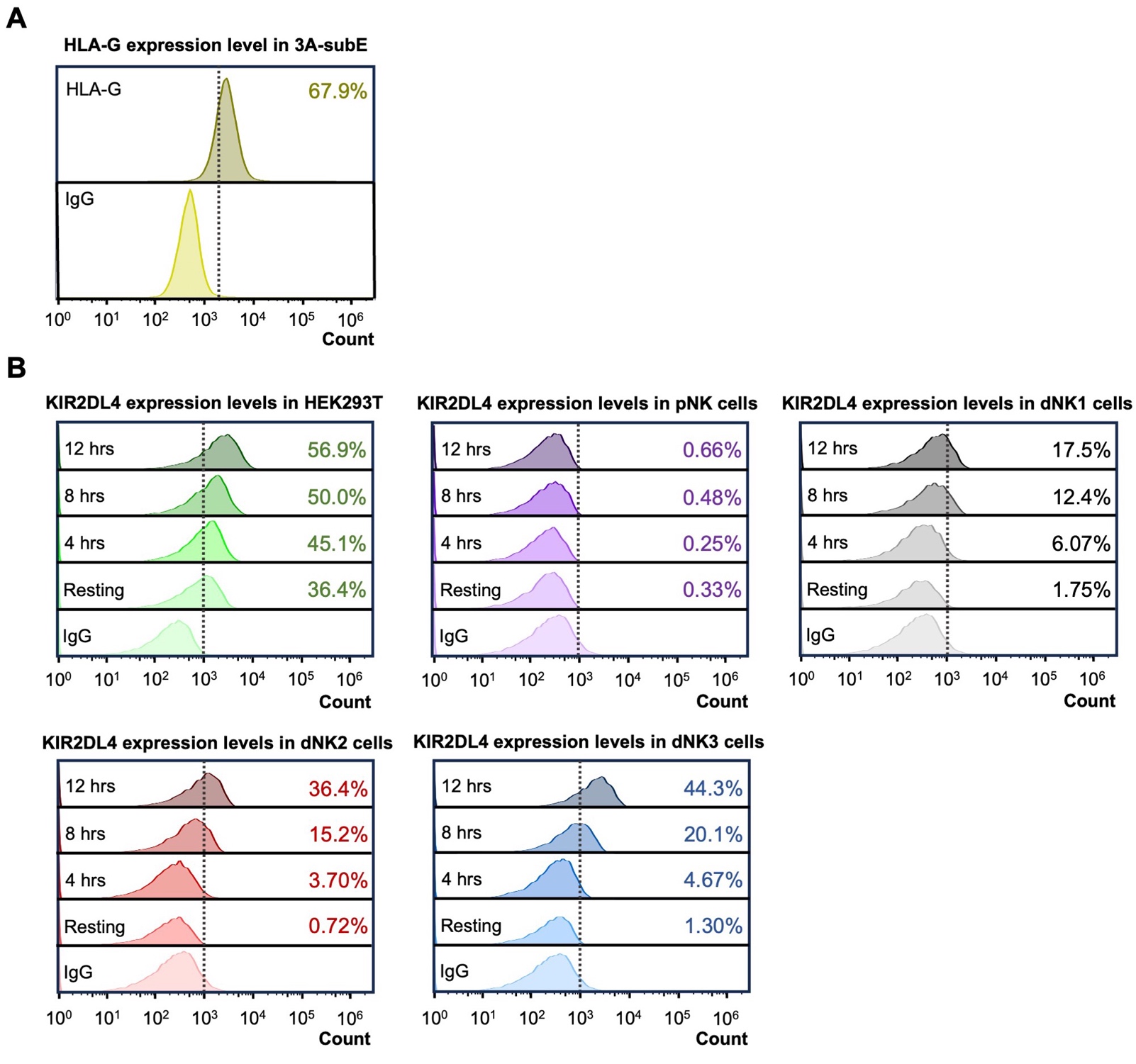


**Supplementary Figure 7 ⏐ HLA-G and KIR2DL4 expression levels in trophoblasts and dNK2 cells.**

**(A)** Expression levels of HLA-G on trophoblasts. **(B)** Measurement of KIR2DL4 expression levels on HEK293T, pNK cells, and dNK cells after co-culturing with trophoblasts at 0 (resting), 4, 8, and 12 hours.


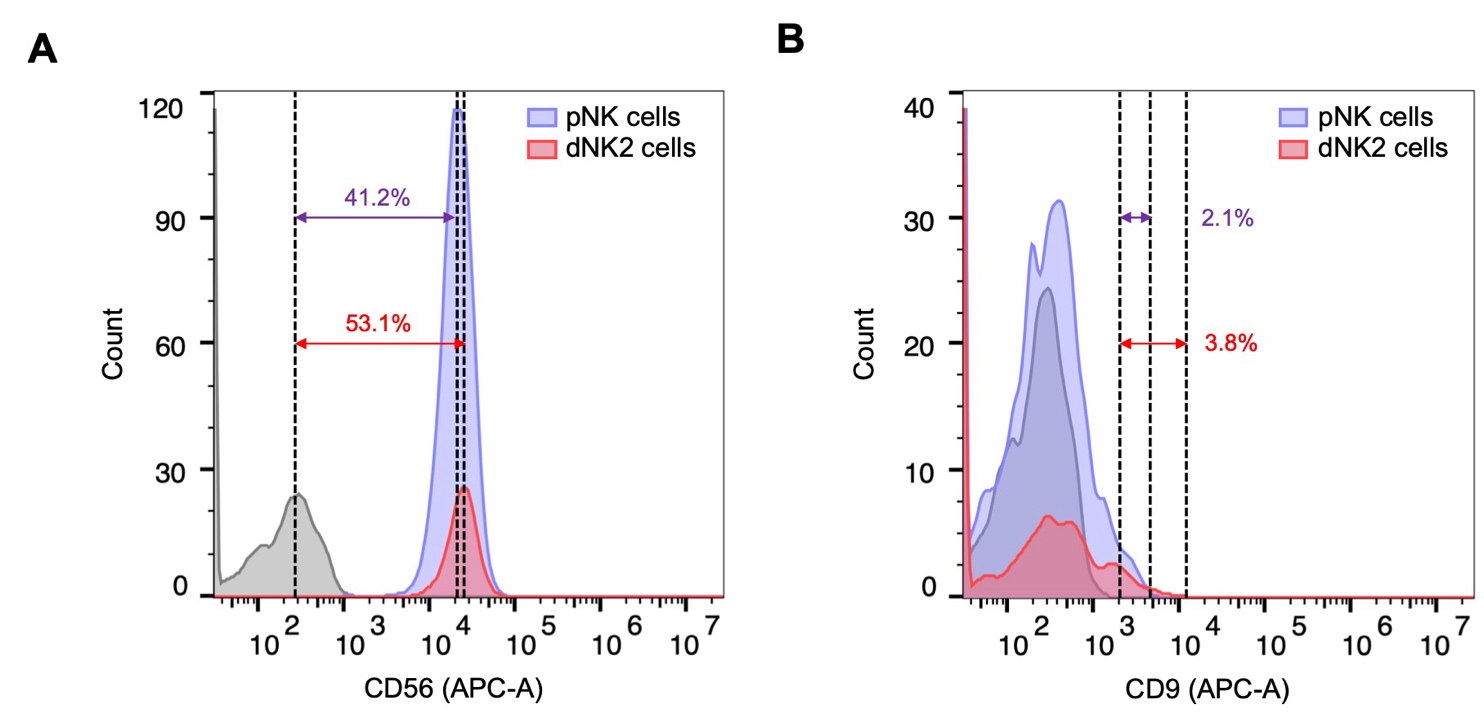


**Supplementary Figure 8 | Characterization of dNK-like cells (dNK2 cells) derived from pNK cells through co-culture with De-MD-MSCs.**

**(A)(B)** Flow cytometry analysis of CD56 and CD9 expression in pNK and dNK2 cells. Gray represents IgG negative control expression levels, purple represents pNK cell expression levels, and pink represents dNK2 cell expression levels.

**Supplementary tables**

**Supplementary Table 1. Diagnostic criteria for PAS patients in this study.**

| **Patient** | **Age** | **Gestational age** | **PAS type** | **Diagnostic criteria** |
| --- | --- | --- | --- | --- |
| PA-MSC-1 | 44 | 34 | Placenta increta  (FIGO grade 2 PAS) | 1. Sonographic findings: Loss of clear zone, and myometrial thinning, presence of abnormal placental lacuna, bladder wall interruption, subplacental and uterovesical hypervascularity, and bridging vessels.  2. Intraoperational findings: Details of findings encountered during the surgical procedure.  3. Pathological findings: Presence of nests of intermediate trophoblasts within myometrial fibers. |
| PA-MSC-3 | 35 | 34 | Placenta percreta  (FIGO grade 3 PAS) | 1. Ultrasound and MRI diagnostic criteria: Loss of the clear zone, myometrial thinning, irregular intraplacental lacuna, subplacental and uterovesical hypervascularity, and the presence of a dark band on MRI.  2. Intraoperational findings: Villous tissue visibly breaching the serosa.  3. Pathological findings: Microscopic examination reveals tissue fragments comprising placental and smooth muscle tissues, with gestational villi in direct contact with the myometrium, consistent with placenta accreta spectrum. Additionally, areas of hemorrhage and infarcted villi are observed. Clinical correlation with patient history is recommended. |
| PA-MSC-4 | 41 | 33 | Placenta percreta  (FIGO grade 3 PAS) | 1. Ultrasound and MRI diagnostic criteria: Loss of clear zone, myometrial thinning, irregular intraplacental lacunae, subplacental and uterovesical hypervascularity, and the presence of a dark band on MRI.  2. Intraoperational findings: Villous tissue visibly present over the serosa.  3. Pathological findings: Microscopic examination reveals villous tissue invading through the clinically identified serosa, consistent with a diagnosis of placenta percreta. |

**Supplementary Table 2. Human primer sequences for RT-qPCR**

| **Gene** | **Primer sequences** | |
| --- | --- | --- |
| Human 18srRNA | F | CGC CGC TAG AGG TGA AAT TCT |
|  | R | CGA ACC TCC GAC TTT CGT TCT |
| Human CRIPLD2 | F | ACG GAC GAG ATG AAT GAG GTG G |
|  | R | GGT GTC ACA TCT GAC GAC TTG G |
| Human FKBP5 | F | GCG AAG GAG AAG ACC ACG ACA T |
|  | R | TAG GCT TCC CTG CCT CTC CAA A |
| Human IGFBP1 | F | TTTTACCTGCCAAACTGCAACA |
|  | R | CCCATTCCAAGGGTAGACGC |
| Human IGFBP4 | F | ACC CAC GAG GAC CTC TAC ATC A |
|  | R | CAC ACC AGC ACT TGC CAC GCT |
| Human IL-15 | F | AAC AGA AGC CAA CTG GGT GAA TG |
|  | R | CTC CAA GAG AAA GCA CTT CAT TGC |
| Human PRL | F | GAG GAG CAA ACC AAA CGG CTT C |
|  | R | AAG GCG AGA CTC TTC ATC AGC C |
| Human PRLR | F | CAT GGT GAC CTG CAT CTT TCC G |
|  | R | GTG GGA GGA AAG TCT TGG CAT C |
| Human REN | F | GAA GAG GCT GAC ACT TGG CAA C |
|  | R | GCA CCC AAA CAT TGG ACG AAC C |
| Human SCARA5 | F | GCT GAA CCT GTG TGA GGA TGG T |
|  | R | TCA GGA AGA CCA GCA GGT AGA G |

**Supplementary Table 3. Antibodies for immunohistochemistry and flow cytometry**

| **Antibodies** | **Brand** | **Catalog number** | **Applications** |
| --- | --- | --- | --- |
| Recombinant Alexa Fluor® 488 Anti-Estrogen Receptor alpha antibody | abcam | ab194150 | FC/ IHC |
| Recombinant Alexa Fluor® 647 Anti-Progesterone Receptor antibody | abcam | ab270198 | FC/ IHC |
| Human SCARA5 Alexa Fluor® 488-conjugated Antibody | R&D | #FAB4900G | FC/ IHC |
| Human Prolactin Capture Antibody | R&D | Part #842242 | IHC |
| Human Prolactin Detection Antibody | R&D | Part #842243 | IHC |
| Human IGFBP1 Capture Antibody | R&D | Part #840402 | IHC |
| Human IGFBP1 Detection Antibody | R&D | Part #840403 | IHC |
| FITC anti-human CD3 | Biolegend | 317306 | FC |
| APC anti-human CD56 | eBioscience | 17-0567-42 | FC/ IHC |
| APC anti-human CD9 | Biolegend | 312107 | FC/ IHC |
| PE anti-human CD16 | Biolegend | 302007 | FC/ IHC |
| PerCP anti-human CD49a | Biolegend | 328321 | FC/ IHC |
| APC anti-human CD158d (KIR2DL4) Antibody | BioLegend | 347008 | FC |
| Alexa Fluor® 488 anti-human HLA-G Antibody | BioLegend | 335917 | FC |
| Recombinant Alexa Fluor® 488 Rabbit IgG | abcam | ab199091 | FC |
| Recombinant Alexa Fluor® 647 Rabbit IgG | abcam | ab199093 | FC |
| Mouse IgG2B Alexa Fluor® 488-conjugated Isotype Control | R&D | #IC0041G | FC |
| APC anti-mouse IgG1 Antibody | BioLegend | 406609 | FC |
| Alexa Fluor® 488 Mouse IgG2a, κ Isotype Ctrl Antibody | BioLegend | 400233 | FC |
